# Management of Small Bowel Perforation by a Bizarre Foreign Body in a 55-Year-Old Woman

**DOI:** 10.1155/2018/2781353

**Published:** 2018-09-12

**Authors:** Francesca D'Auria, Vincenzo Consalvo, Antonio Canero, Maria Russo, Carmela Rescigno, Domenico Lombardi

**Affiliations:** ^1^General Surgery, Università degli Studi di Salerno, Via Giovanni Paolo II, 132, 84084 Fisciano, Italy; ^2^Clinique Clementville, rue de Clementville, Montpellier, France; ^3^Azienda Ospedaliera Universitaria San Giovanni di Dio e Ruggi d'Aragona, Via San Leonardo, 1, Salerno, Italy

## Abstract

**Introduction:**

Ingestion of foreign bodies including dentures, fishbone, screw, and/or surgical devices can be a cause of morbidity, and it rarely could be fatal.

**Presentation of Case:**

We present the first hitherto reported case of mussel shell ingestion, which caused acute abdominal pain in a 55-year-old woman. The shell pierced ileal loops, and it was found in the abdominal cavity.

**Discussion:**

The accidental or voluntary ingestion of a foreign body is an uncommon event compared to the other causes of bowel perforation. It is fundamental to immediately remove the intestinal fluid, repair the tear, and prevent sepsis, because each delay in diagnosis can lead to a worst outcome.

**Conclusion:**

In case of bowel perforation, it important for surgeons, who are dealing with these acute care patients, to be aware of different designs and constructions of possible foreign bodies, in order to be prepared to deal with different possible scenarios and be able to manage them properly.

## 1. Introduction

Foreign body ingestion is a commonly seen accident in emergencies [1–3]. It may accidentally occur in children or intentionally in psychiatric patients. In the 90% of ingested foreign bodies, they pass through the gastrointestinal tract without complications as punching or obstruction, but in the 10% of the case, they required a surgical removal [4]. Ingestion of foreign bodies including dentures, fishbone, screw, and/or surgical devices can be a cause of morbidity and mortality [5–7]. We report the first case of mussel shell ingestion which caused acute abdomen.

## 2. Ethical and Administrative Information

The patients gave her consent to the publication of scientific data. The authors declare that there will not be any communication to the third party for the respect of her privacy. This study was written respecting the ethical principles for medical research involving human subjects (Declaration of Helsinki). This article was written according to SCARE 2016 guidelines on case report writing. Since other few cases have been reported, it was not necessary to publish on a public registry.

## 3. Case Report

**Figure 1:**
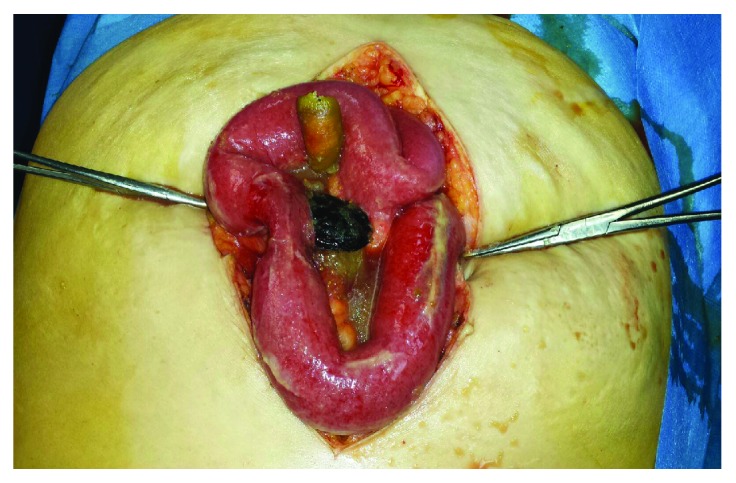
View of the abdomen at the opening of the peritoneum.

**Figure 2:**
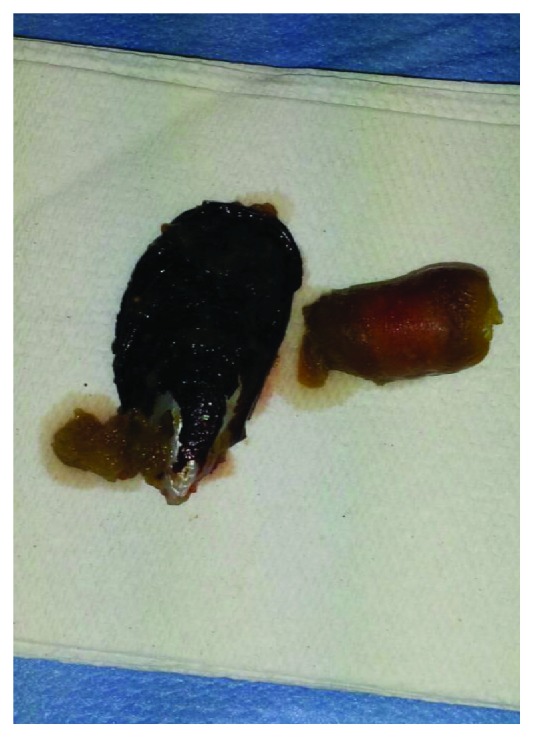
Complete extraction of the foreign body.

A 55-year-old Caucasian obese woman (body mass index = 35) was admitted to Surgical Department of our institution for acute abdominal pain. Her past medical history was negative for previous gastrointestinal disease or surgery. She was on medical therapy for hypertension, type II diabetes, and minor depression. Glasgow coma scale was 15. She referred an increasing acute abdominal pain risen 5 hours ago after a fish-based dinner. She has showed an acute diffuse peritonitis. White blood cell count was 32.000 U/*μ*L, with neutrophilia (90%); other blood tests were in normal range. Body temperature was 39.2°C. Electrocardiogram showed sinus rhythm with 92 heart rate. Chest X-ray was normal. Abdominal X-ray showed free subdiaphragmatic air. CT scan confirmed the suspicion of small bowel perforation because of the finding of free fluid in the abdomen and an inhomogeneous mass in the small bowel. A nasogastric tube was placed, and it drained 50 mL of biliogastric material. Because of her status, she was immediately ran to the theater for exploratory laparotomy under general anesthesia and oral intubation. Although each clinical finding suggested a colonic or caecum perforation, during the systematic exploration of the bowel loops, surgeons found free intestinal fluid in the abdomen, fecal peritonitis, and (at 60–70 from ileocaecal valve) a 3 cm linear tear of the ileum which was caused by the curve edge of a shell mussel (Figure 1). The foreign body was completely extracted from the lumen through the hole (Figure 2), and the breach was sutured with simple double-strand stitches of polyglactin 3/0 parallel to the bowel tearing. Abdominal cavity washing was carried out with 2 liters of saline. Two drains were placed on suction for 24 hours. Antibiotic therapy (ciprofloxacin, meropenem, and metronidazole) and nil by mouth regimen were started. Patient was admitted in Intensive Care Unit for 12 hours, the weaning from the ventilator, and she was discharged at home in healthy status from the ward on the sixth postoperative day. At the 30-day follow-up, the patient was in good clinical condition, surgical wounds were completely sealed, blood tests were normal, and bowel function was recovered.

## 4. Discussion

The accidental or intentional ingestion of foreign bodies including dentures, fishbone, screw, magnets, lithium batteries, and/or surgical devices can be a cause of morbidity, but mortality rates have been extremely low. As a matter of facts, a compilation of multiple studies including 2 large series report no deaths in 852 adults and 1 death in 2206 children; mortality rate is extremely low [1, 2]. In the 90% of ingested foreign bodies, they pass through the gastrointestinal tract without complications as punching or obstruction, but in the up to 10% of the case, they required an endoscopic or open/laparoscopic surgical removal [4, 5]. The foreign body ingestion is a commonly seen in emergency setting [7, 8]. It may accidentally occur in children, elderly, edentulous patients, patients with neurological disorders, and addicted patients or intentionally in psychiatric patients or prisoners. A slight prevalence in male is documented [1–3]. Any foreign body that remains in the tract may cause obstruction, perforation or hemorrhage, and fistula formation. The most common sites of perforation are the ileocaecal junction and sigmoid colon. Other potential sites are the duodenojejunal flexure, appendix, colonic flexure, diverticula, and the anal sphincter [8]. Exploratory laparotomy has been the main treatment for patients requiring surgery. However, surgeons are trying to routinely use laparoscopy in the emergency setting aiming reduction in the length of stay and for esthetical purpose [5]. In the diagnosis management, CT scan is a mandatory requirement to orient the endoscopic or surgical management. In case of bowel perforation, the correct management should be the precocious removal of the foreign body, the suture of the breach, and the toilette of the abdominal cavity in order to reduce the septic risk and restore bowel function [8, 9].

## 5. Conclusion

Inflammatory bowel disease, appendicitis, diverticula, gallstone migration, cancer, endometriosis, trauma, infection, autoimmune disease, drugs, vasculopathy [10–13], and foreign bodies can cause an acute bowel perforation. It is fundamental to immediately remove the intestinal fluid, repair the tear, and prevent sepsis. Each delay in diagnosis can lead to a worst outcome. Because the accidental or voluntary ingestion of a foreign body is uncommon compared to the other causes of bowel perforation, it is important to remember that it is a possible event which deserves to be contemplated in the list of causes to be investigated in case of free air in abdomen.

## Additional Points

*Highlights*. A foreign body ingestion is an uncommon cause of bowel perforation. It may accidentally occur in children or intentionally in psychiatric patients. Differential diagnosis is mandatory, i.e., IBD, diverticula, vasculopathy, etc. It is fundamental to remove intestinal fluid, repair bowel tear, and prevent sepsis. Each delay in diagnosis can lead to a worst outcome.
